# Evaluation of ASA, SORT, and ACCI Scores in Predicting the Need for Postoperative Intensive Care Unit Admissions After Hip Surgery

**DOI:** 10.4274/TJAR.2025.241708

**Published:** 2025-07-24

**Authors:** Neslihan Gezer, Lütfiye Pirbudak, Elzem Şen, Ayşe Mızrak

**Affiliations:** 1 Gaziantep University Faculty of Medicine Department of Anaesthesiology and Reanimation, Gaziantep, Türkiye

**Keywords:** ACCI, ASA, hip surgery, ICU admission, SORT

## Abstract

**Objective:**

We aimed to investigate the effectiveness of the American Society of Anesthesiologists (ASA), the Surgical Outcome Risk Tool (SORT), and age-adjusted Charlson Comorbidity Index (ACCI) scores in determining the requirement for intensive care unit (ICU) admissions in patients aged 65 years and older who underwent hip surgery.

**Methods:**

The study population consisted of 450 patients who underwent orthopedic hip surgery. The patients who were admitted to the ICU were either monitored in the postoperative ICU (Group 1) or transferred to the ward (Group 2). SORT and ACCI scores of all patients were recorded.

**Results:**

The patients of Group 1 were significantly older than those in Group 2. SORT scores of both groups were comparable. The diagnostic sensitivity and specificity of ACCI scores were determined as 42.1% and 70.8%, respectively.

**Conclusion:**

As a conclusion, ACCI scores can predict the need for ICU admissions in patients undergoing hip surgery. Besides, the traditionally used ASA scores are generally higher in this patient group. Determinative criteria for predicting the need for ICU admissions include older age of the patients, presence of comorbidities as hypertension and diabetes mellitus, as well as a long preoperative waiting period.

**Main Points**

• It is important to identify preoperative risk factors for unplanned admission to the intensive care unit to improve the allocation of medical resources and the quality of care.

• The Surgical Outcome Risk Tool is a simple model that predicts 30-day mortality rates before surgery using six routinely collected data elements.

• The Charlson Comorbidity Index (CCI) has been proven to predict postoperative complications and one-year mortality.

• Additionally, the age-adjusted CCI can be used to determine the need for intensive care in patients undergoing hip surgery.

## Introduction

Expectedly, the number of hip fractures will reach 4.5 million by the year 2050 as a result of an increase in the aging population worldwide.^1^ If not contraindicated, hip fracture cases are usually treated surgically due to the high morbidity and mortality of conservative treatment modalities. However, patients undergoing surgery may face certain challenges. Despite still significant number of patients needing to be admitted to the intensive care units (ICUs) following hip surgeries, a multidisciplinary geriatric approach is employed. Due to the lack of guidelines for admissions to the postoperative ICUs, hospital organizations implement different strategies. Therefore, recognising preoperative risk factors for unplanned admissions to the ICUs is crucial to improve both allocation of medical resources and quality of care.^2^

In 1963, the American Society of Anesthesiologists (ASA) established a numerical system to assess the perioperative risk and physical health status of patients during anaesthesia and surgical procedures. Higher ASA scores were associated with increased postoperative complications, admissions to the ICU, and higher mortality rates. ASA scores are routinely determined subjectively by the anaesthesiologist before anaesthesia and can be easily found in medical records.^3^ Surgical Outcome Risk Tool (SORT) is a simple model designed to preoperatively predict 30-day mortality rates, using six routinely collected data elements.^4^

The Charlson Comorbidity Index (CCI) has been shown to predict postoperative complications and one-year mortality rates in patients undergoing surgical treatment. Furthermore, since a patient’s age is closely associated with prognosis, an age-adjusted CCI (ACCI) was introduced into clinical practice in 1994. A CCI score was created by adding one point for every decade of age increase for patients aged 40 years or older, resulting in the age-adjusted CCI score.^5^

In this study, we aimed to investigate the effectiveness of ASA, SORT, and ACCI scores in determining the requirement for ICU admissions in the preoperative evaluation of patients aged 65 and older who underwent hip surgery. The study’s hypothesis is that SORT, a risk stratification tool comprising six preoperative variables, is superior to ACCI and ASA scores in predicting ICU admission.

## Methods

This retrospective case-control study was conducted after the approval of the Gaziantep University Clinical Research Ethics Committee (date: 25.05.2022, approval no.: 2022/55) and in accordance with the ethical principles of the Declaration of Helsinki on ethical principles for medical research involving human subjects released by the World Medical Association in 2013. The study was organized after obtaining written consent from a total of 450 patients who underwent orthopedic hip surgery at Gaziantep University Şahinbey Research and Practice Hospital between April 1, 2016 and August 1, 2021. Patients aged ≥65 years with ASA scores I-IV were included in the study. Patients who underwent revision surgery, those who underwent multi-trauma surgery, those followed up in the ICU during the preoperative period, and cases with missing data were not included in the study.

Pre- and post-operative patients’ data, derived from hospital records, were evaluated. The patients admitted to ICU were either monitored in the postoperative ICU (Group 1) or transferred to the ward without the need for postoperative ICU monitoring (Group 2). Information concerning the age, gender, body mass index (BMI), and ASA scores of the patients; type of surgery; smoking status; type of anaesthesia applied; preoperative period lasting from admission to the start of the surgery; duration of surgery; and comorbidities (hypertension, diabetes mellitus, coronary artery disease, chronic obstructive pulmonary disease, chronic kidney disease, osteoporosis, malignancy, neurological diseases) were recorded from patients’ medical files.

The SORT scores of the cases were obtained from the http://www.sortsurgery.com/ website (accessed on March 15, 2022) after a detailed retrospective scan of the patients’ computerized database. The ACCI index and relevant parameters were electronically entered into the https://www.mdcalc.com/charlson-comorbidity-index-cci#use-cases website (accessed on March 15, 2022), and necessary calculations were made.

### Statistical Analysis

Assuming a medium effect size (Cohen’s d = 0.5) with an alpha level of 0.05 and a target power of 90%, our analysis indicated a minimum of 85 patients per group, yielding a total sample size requirement of approximately 170 participants.

The normal distribution of numerical variables was tested using the Shapiro-Wilk test. For variables that followed a normal distribution, the Independent Samples t-test was used. For non-normally distributed variables, the Mann-Whitney U test was applied. The chi-square test was used to compare categorical data between groups. receiver operating characteristic (ROC) curve analysis was conducted to determine the cut-off points for ACCI, SORT, and ASA. Version 22.0 of the Windows statistical package program was used for the analyses, and *P* < 0.05 was considered statistically significant.

## Results

The data on 190 patients, in Group 1, and 260 patients, in Group 2, were comparatively reviewed. Any statistically significant differences were not observed between Groups 1, and 2 in terms of distribution of male/female patients (*P*=0.09), mean BMIs (*P*=0.11), ASA physical status scores (*P*=0.31), the number of smokers (*P*=0.09), types, and duration of surgical interventions (*P*=0.34 vs *P*=0.039). However, the patients of Group 1 (mean age: 82.14±8.96 years) were significantly older compared to Group 2 (mean age: 71.61±8.06 years) (*P*=0.03), and time to surgery was statistically significantly longer in Group 1 patients (*P *< 0.05). The groups were similar concerning the type of anaesthesia used (*P*=0.51) (Table 1).

As is seen in Table 2, statistically significant differences existed between Groups 1 and 2 in terms of the presence of hypertension (*P*=0.03) and diabetes mellitus (*P*=0.03). While mortality rates were statistically significantly higher in Group 1 (*P*=0.01) (Table 2), (complete the sentence with an independent clause).

SORT scores of both groups were similar (*P*=0.85). The ACCI scores were statistically higher in Group 1 (*P*=0.02) (Table 3).

**Figure 1:**
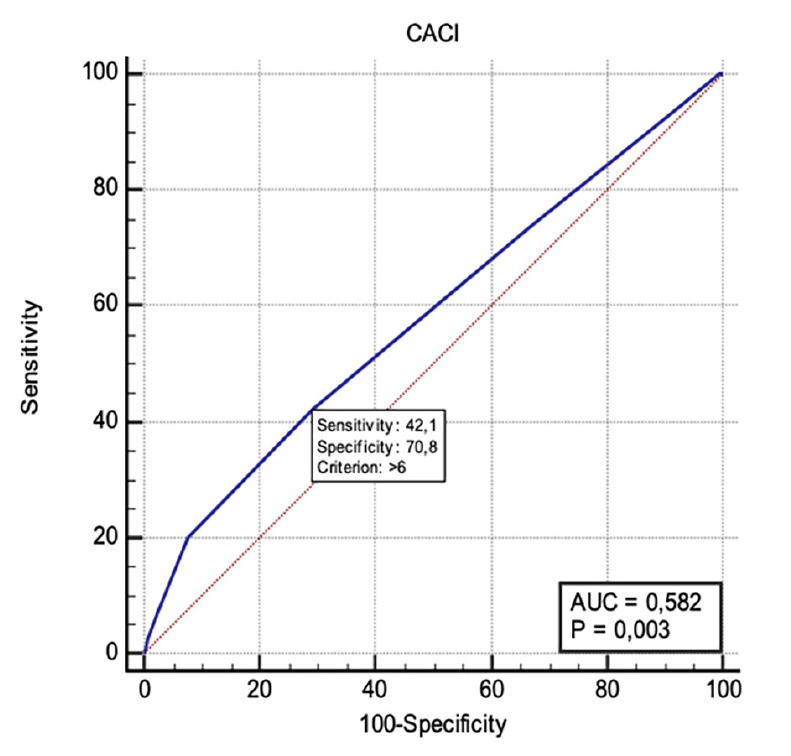
ROC curve analysis for ICU admission prediction based on ACCI score. ROC, receiver operating characteristic; ICU, intensive care unit; ACCI, age-adjusted Charlson Comorbidity Index.

ROC curve analysis was performed to evaluate the predictive value of three scoring systems for ICU admission. ACCI showed a statistically significant predictive ability with an AUC of 0.582 [95% confidence interval (CI): 0.535-0.628, *P*=0.003]. The optimal cut-off value was determined to be >6, with a sensitivity of 42.11% and a specificity of 70.77% (Figure 1).

**Figure 2:**
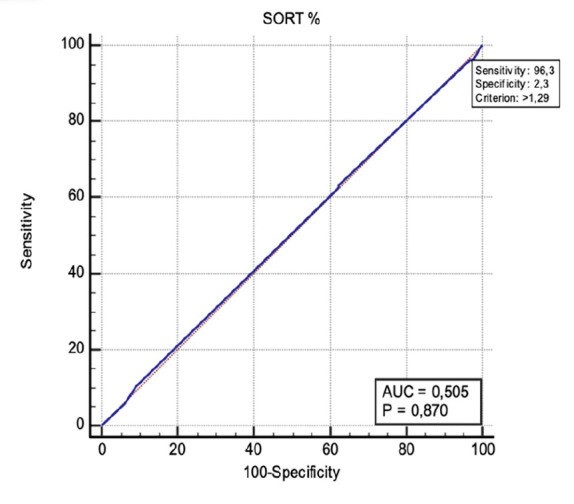
ROC curve analysis for ICU admission prediction based on SORT score. ROC, receiver operating characteristic; ICU, intensive care unit; SORT, Surgical Outcome Risk Tool.

The SORT score demonstrated poor predictive performance, with an AUC of 0.505 (95% CI: 0.457-0.552, *P*=0.870), indicating no significant discrimination between ICU and non-ICU patients. Despite the high sensitivity (96.32%), specificity was notably low (2.31%) at the cut-off value of >1.29 (Figure 2).

**Figure 3:**
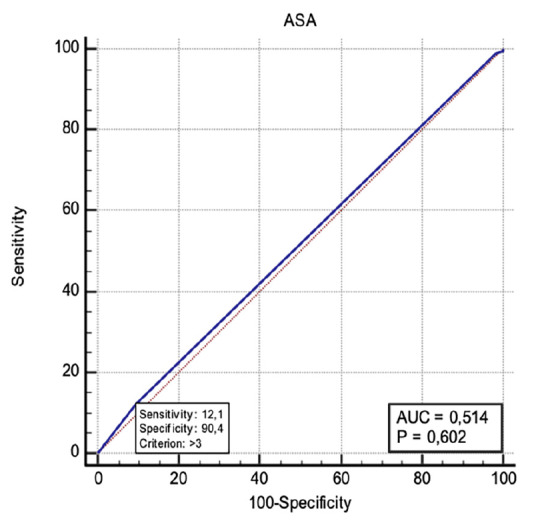
ROC curve analysis for ICU admission prediction based on ASA score. ROC, receiver operating characteristic; ICU, intensive care unit; ASA: American Society of Anesthesiologists; AUC, area under the curve.

Similarly, the ASA classification yielded an AUC of 0.514 (95% CI: 0.467-0.561, *P*=0.602), also reflecting limited discriminatory power. The cut-off point >3 provided a sensitivity of 12.11% and a specificity of 90.38% (Figure 3).

## Discussion

In this study, we aimed to evaluate ASA, SORT, and ACCI scores in predicting the postoperative ICU admissions of the patients who had undergone orthopedic hip surgery. Postoperative ICU follow-up of critical patients remains an important part of the treatment process. Risk classification tools help clinicians provide more accurate information to patients, which also guide perioperative care decisions. Simple and cost-effective risk-scoring tools will become more widely used, especially with the growing availability of mobile digital devices.

Surace et al.^6^ found that longer operation time was associated with a greater risk of readmissions, reoperations, surgical site infections, systemic complications, and blood transfusions. They identified that the rate of these complications increased when the duration of surgery exceeded 75-80 minutes. They also found a relationship between venous thromboembolic complications and surgeries lasting approximately 90 to 100 minutes. In this study, the duration of surgery in both groups was similar, which could be attributed to the limited number of cases in our study and the fact that postoperative complications were not investigated in the late postoperative period. Most available data point to the potential benefits of a comprehensive medical approach prioritizing regional anaesthesia for patients and the healthcare system.^7^ In our study, regional anaesthesia was found to be the most commonly used type of anaesthesia for hip surgeries and was applied at similar rates in both groups.

Older age, higher ASA class, and duration of surgery longer than 4 hours have been found to be associated with a higher probability of unplanned postoperative admissions to the ICU.^8^ Although there was no significant difference between the groups in our study, we observed that the mean age of the group with patients deemed appropriate for postoperative ICU admission was significantly higher. A short time interval between admission and surgery is considered ideal for geriatric hip fractures. Despite the importance of shortening the time to surgery for reducing mortality rates after hip fractures, no significant differences were found when considering 48 hours as a critical cut-off point for mortality.^9^ In our study, the time to surgery was longer in the group of patients admitted to the ICU, and mortality rates were higher in these patients. Comorbidities are often cited as risk factors for mortality or morbidity assessments in ICU or ward patients.^10^ Similarly, in the present study, the statistically higher number of patients with hypertension and/or diabetes mellitus was postoperatively admitted to ICUs.

The positive correlation between ASA scores and postoperative mortality rates was first published in 1970 and has been recently emphasised in a large prospective study that compared more than 700,000 patients undergoing elective and emergency procedures, indicating the importance of ASA scores in predicting mortality within 48 hours after surgery.^11^ In their retrospective cohort study, Park et al.^12^ observed higher ICU admission rates and prolonged hospital stay in the ASA III group compared to ASA I and II groups of patients who had undergone laparoscopic colorectal surgeries. Since distribution of ASA scores between both groups was comparable in our study, we thought that ASA scoring system may not provide meaningful information in determining the need for ICU admissions.

It has been suggested that SORT scores contribute to the identification of high-risk patients, thus serving as a useful tool not only for resource planning but also for preoperative assessment, informed consent, and shared decision-making processes. Therefore, SORT is expected to have a leading role in routine clinical practice among preoperative mortality risk assessment tools in terms of evaluating and contributing to improving patient outcomes.^13^SORT has been shown to predict the risk of postoperative morbidity in major elective surgery when used preoperatively.^4^ Aboosalih et al.^14^ concluded that SORT can be used to identify high-risk patients and assess the need for intensive care admissions. SORT has a lower predictive value in evaluating the need for postoperative admissions to ICU, as we also investigated in our study. We also considered that there was no statistically significant difference ICU admission rates between postoperative ICU and orthopedic ward patients due to the small sample size of our study.

CCI has been used in many studies to predict postoperative mortality in patients undergoing surgery. In a study of 497 patients undergoing surgical resection for pancreatic cancer, Dias-Santos et al.^15^ found that a CCI score of >4 predicted prolonged hospital stay. In another study, Zhan et al.^16^ revealed a significant relationship between CCI scores and admissions to ICU in patients undergoing thoracic aortic aneurysm surgery. St-Louiset al.^17^ showed that ACCI could be a reliable predictor of postoperative outcomes in emergency general surgery patients. They also found that ACCI could be a predictor of 30-day mortality. Therefore, considering the simplicity of the CCI model, it has been observed to be a good option for predicting perioperative mortality. In our study, we also found that the ACCI scores could be a valuable prognostic tool in predicting the need for postoperative admissions to the ICU with a sensitivity of 42%, and specificity of 71%.

### Study Limitations

The present study was conducted at a single center. To confirm our results, we need multicenter studies with more patients.

## Conclusion

In conclusion, we believe that ACCI can be a determinant in predicting the need for ICU admissions in patients undergoing hip surgery. In this patient group with generally higher ASA scores, the determinative criteria for predicting the need for ICU admissions are older age, the presence of comorbidities such as hypertension and diabetes mellitus, and a prolonged preoperative waiting period. To demonstrate the effectiveness of the SORT index, which encompasses all determinants such as the patient’s age, ASA physical status, comorbidities, and surgical characteristics, in predicting the need for ICU, further clinical studies should be conducted with a higher number of patients.

## Ethics

**Ethics Committee Approval:** This retrospective case-control study was conducted after the approval of the Gaziantep University Clinical Research Ethics Committee (date: 25.05.2022, approval no.: 2022/55).

**Informed Consent:** Written consent was obtained from patients.

## Figures and Tables

**Table 1. Demographic and Clinical Features of the Patients:** 

-	**Group 1** **n = 190**	**Group 2** **n = 260**	***P* value**
**Male/female n (%)**	82 (43.2%)/108 (56.8%)	92 (35.4%)/168 (64.6%)	0.09
**Age (years) (mean ± SD)**	82.14±8.96	71.61±8.06	0.03*
**BMI (kg/m^2-1^) (mean ± SD)**	26.04±4.03	25.41±4.03	0.11
**ASA I/II/III/IV (n)**	1/1/165/23	0/4/231/25	0.31
**Smokers n (%)**	35 (18.4%)	33 (12.7%)	0.09
**Operation type n (%)** Proximal femur nailing Partial hip replacement Total hip replacement	- 54 (28.4%) 124 (65.3%) 12 (6.3%)	- 62 (23.8%) 186 (71.5%) 12 (4.6%)	0.34
**Time to operation (day) (mean ± SD)**	4.23±3.55	3.46±3.05	0.006*
**Operation duration (minute) (mean ± SD)**	141.24±68.73	150.23±70.59	0.24
**Anaesthesia type n (%)** Spinal anaesthesia Combined spinal epidural anaetshesia Lumbar plexus block General anaesthesia	- 127 (66.8%) 29 (15.2%) 4 (2.1%) 30 (15.7%)	- 189 (72.6%) 29 (10%) 6 (2.3%) 36 (13.8%)	0.51

*Significant at *P *< 0.05.

Pearson’s chi-square test was used for gender, smoking status, operation type, and anaesthesia type. Fisher’s exact test was applied for ASA classification due to small expected cell counts. Quantitative variables (e.g., age, BMI, time to operation, operation duration) were compared using the independent samples t-test or the Mann-Whitney U test, depending on the distribution of the data (tested with the Shapiro-Wilk test).

SD, standard deviation; ASA, American Society of Anesthesiologists; BMI, body mass index.

**Table 2. Comorbidities and Mortality Rates According to the Groups:** 

-	**Group 1** **n (%)**	**Group 2** **n (%)**	***P* value**
**Hypertension**	123 (64.7%)	142 (54.6%)	0.03*
**Diabetes mellitus**	78 (41.1%)	81 (31.2%)	0.03*
**Coronary artery disease**	58 (30.5%)	71 (27.3%)	0.45
**Chronic obstructive lung disease**	34 (17.9%)	34 (13.1%)	0.15
**Other comorbidities** Neurological diseases Osteoprosis Chronic renal failure Malignancy	- 46 (24.2%) 2 (1.1%) 13 (6.8%) 7 (3.7%)	- 43 (16.5%) 6 (2.3%) 11 (4.2%) 10 (3.8%)	0.14
**Mortality**	29 (15.3%)	13 (5%)	<0.01*

*Significant at *P *< 0.05.

Pearson’s chi-square test was used for hypertension, diabetes mellitus, coronary artery disease, and chronic obstructive pulmonary disease. A continuity-corrected chi-square test was used for mortality. Fisher’s exact test was applied for rare comorbidities with low frequencies, including neurological diseases, osteoporosis, chronic renal failure, and malignancy.

**Table 3. Comparison of SORT and ACCI Scores Between Groups:** 

-	**Group I** **(n = 190)**	**Group II** **(n = 260)**	***P* value**
**SORT (%) (mean ± SD)**	5.29±2.87	5.3±2.9	0.85
**ACCI (mean ± SD)**	6.4±1.41	5.98±1.17	0.02*

*Significant at *P *< 0.05, quantitative data were compared using the independent samples t-test.

SORT, Surgical Outcome Risk Tool; ACCI, age-adjusted Charlson Comorbidity Index; SD, standart deviation.
